# Supplementary material for: Mitochondrial impairment activates the Wallerian pathway through depletion of NMNAT2 leading to SARM1-dependent axon degeneration
Source: Neurobiol Dis. Author manuscript; Available in PMC 2021 Oct 6. (PMC7611775; doi:10.1016/j.nbd.2019.104678)
Supplement: Supplementary material [file EMS135867-supplement-Supplementary_material.pdf]

Fig. S1

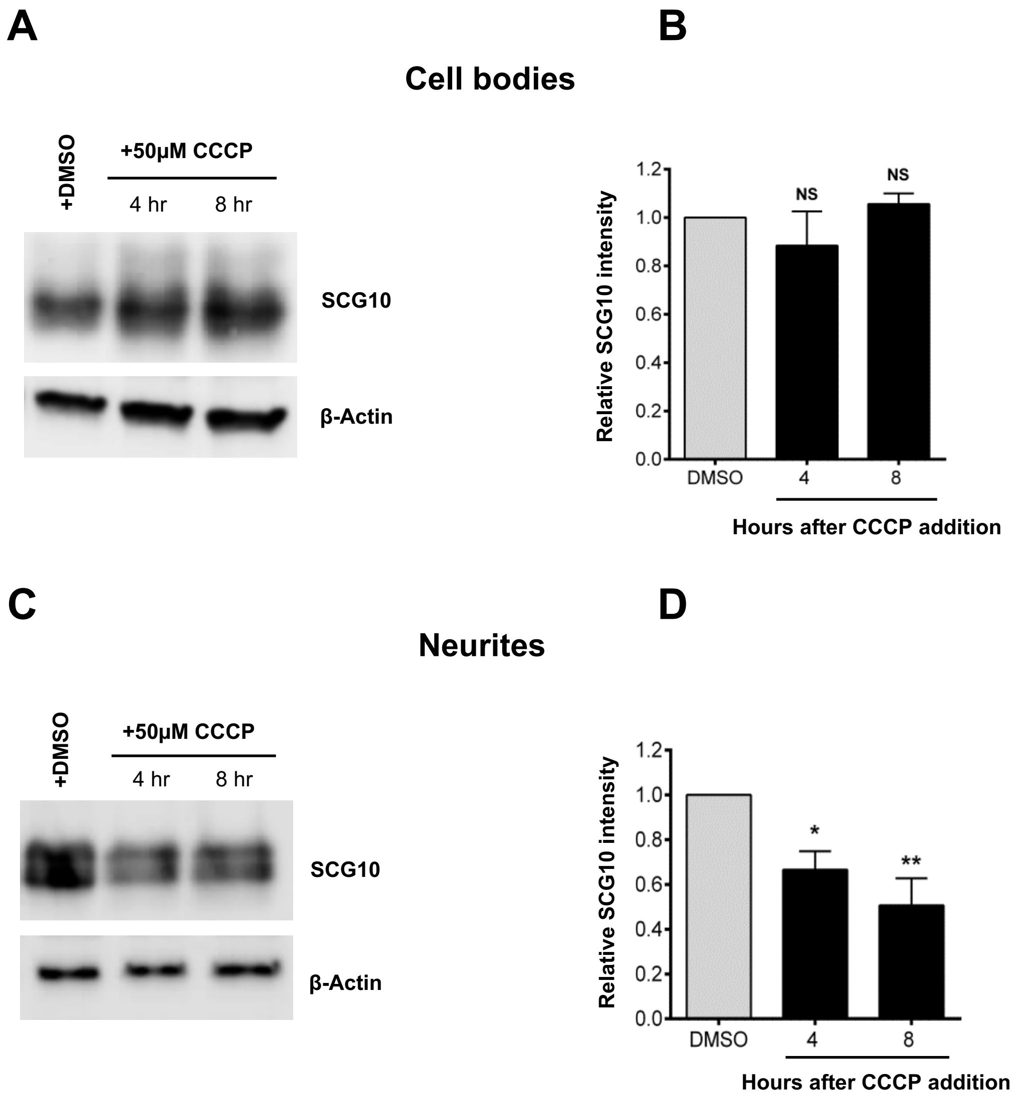

Figure S1.

(A) Representative immunoblot of wild-type SCG cell bodies/ganglia extracts probed for SCG10 and β-actin (loading control) at the indicated time points after CCCP treatment. **(B)** Quantification of normalised SCG10 levels (to β-actin) is shown, with data presented relative to DMSO control (Mean±SEM; n=4; one-way ANOVA followed by Bonferroni post-hoc test; NS, non-significant). **(C)** Representative immunoblots of wild-type SCG neurite extracts probed for SCG10 and β-actin (loading control) at the indicated time points after CCCP treatment. **(D)** Quantification of normalised SCG10 levels (to β-actin) is shown, with data presented relative to DMSO control (Mean±SEM; n=4; one-way ANOVA followed by Bonferroni post-hoc test; **, $p<0.01$; *, $p<0.05$).

Fig. S2

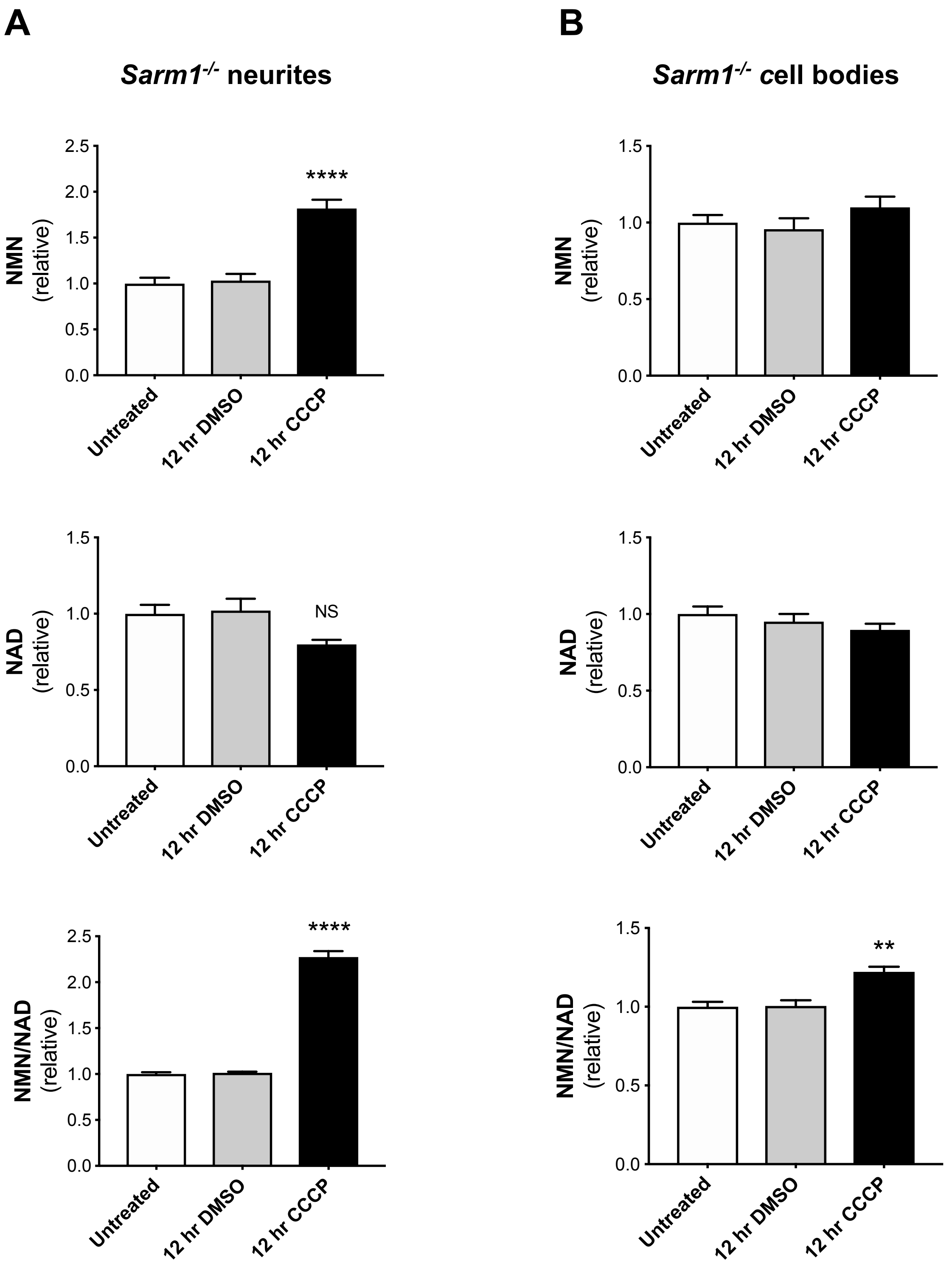

Figure S2.
(A,B) Relative changes in NMN, NAD and NMN/NAD ratios in separate neurite (A) and cell bodies/ganglia (B) fractions from *Sarm1*$^{-/-}$ SCG explant cultures at the indicated time points after CCCP treatment (Mean±SEM; n=5; one-way ANOVA followed by Bonferroni post-hoc test; ****, $p<0.0001$; **, $p<0.01$; NS, non-significant. Statistical significance shown relative to 12 hr DMSO).

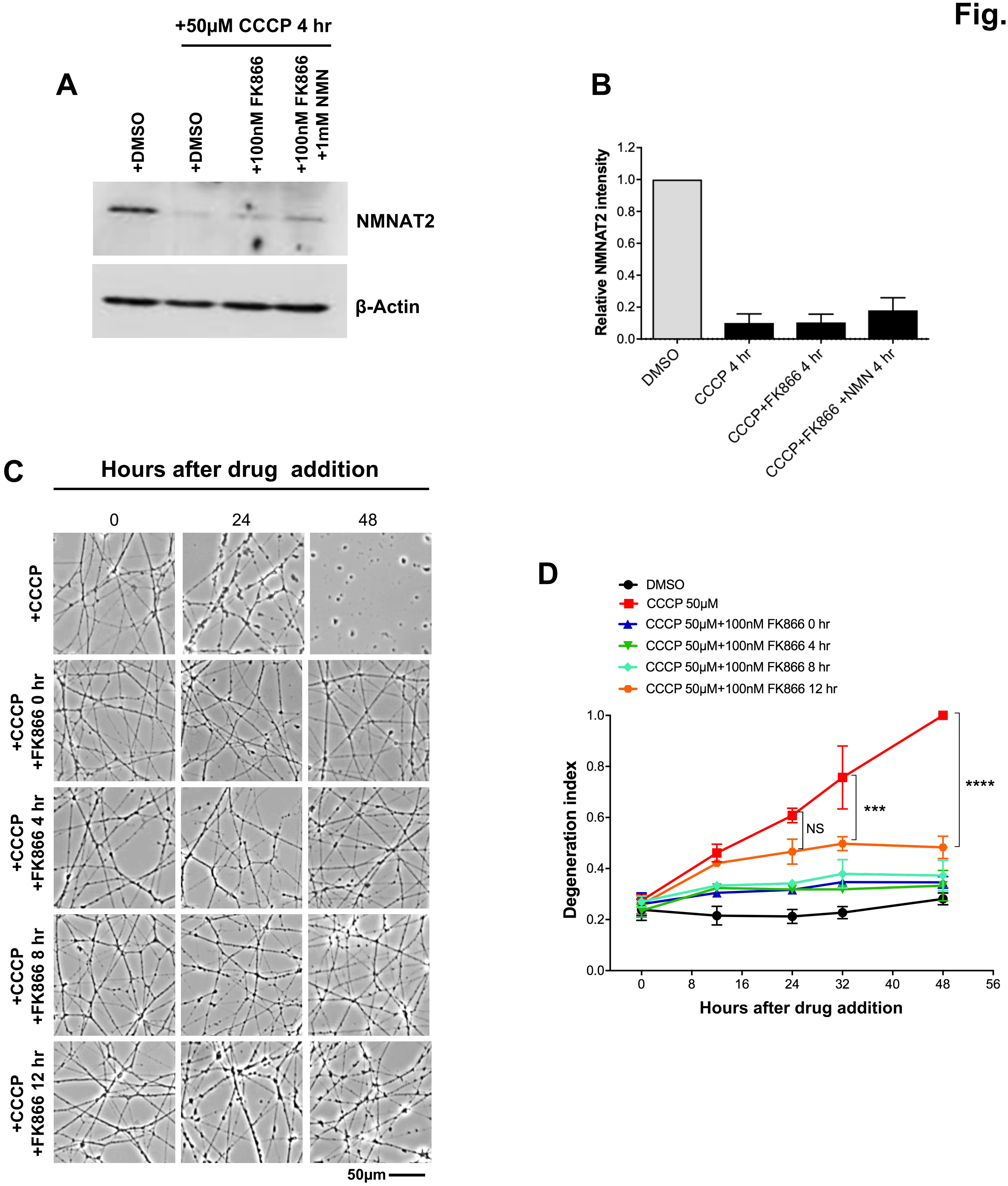

Figure S3.
(A) Representative immunoblot of wild-type SCG neurite extracts probed for NMNAT2 and β-actin (loading control) 4 hr after CCCP, FK866 and NMN treatment. **(B)** Quantification of normalised NMNAT2 and SCG10 levels (to β-actin) is shown, with data presented relative to DMSO control (Mean±SEM; n=2). **(C)** Representative phase contrast images of neurites from wild-type SCG explant cultures at the indicated time points after CCCP treatment. Where indicated, FK866 was added either at the same time of CCCP (0 hr) or 4, 8, 12 hr after CCCP addition. **(D)** Quantification of the degeneration index in experiments described in (B) from 3 fields per sample in 3 independent experiments (Mean±SEM; n=3; two-way ANOVA followed by Tukey post-hoc test; ****, $p<0.0001$; ***, $p<0.001$; NS, non-significant. Statistical significance shown relative to +50μM CCCP).

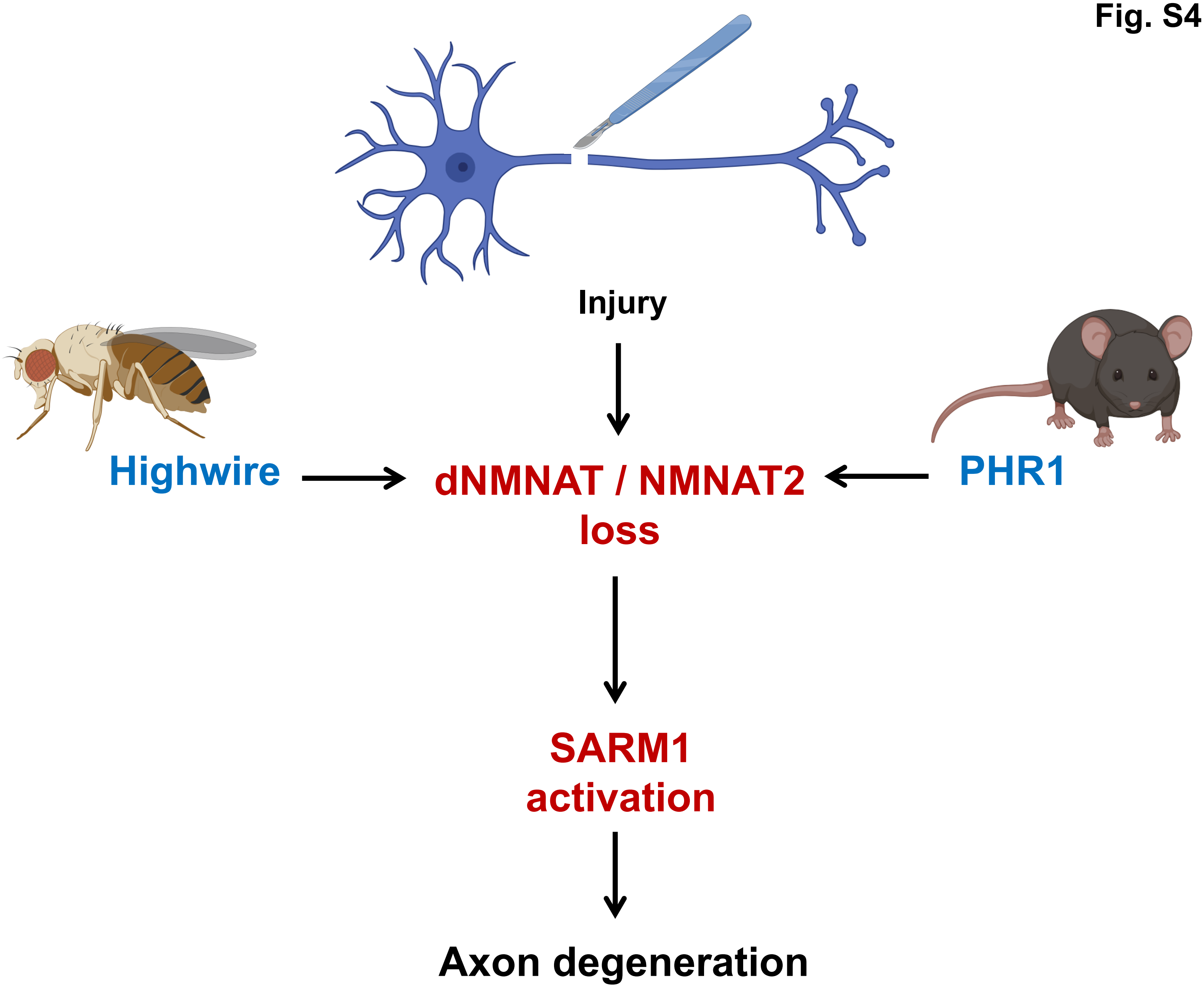

Figure S4.
The Wallerian pathway is evolutionary conserved in flies and mammals. Highwire position in the pathway is shown (‘Created with BioRender’).
